# Differential Use of Depression and Anxiety Medications in Adults With a History of Cancer

**DOI:** 10.1001/jamanetworkopen.2025.27585

**Published:** 2025-08-19

**Authors:** Daniela Miro-Rivera, Ryan A. Norris, Oyomoare L. Osazuwa-Peters, Jillian H. Hurst, Justin M. Barnes, Nosayaba Osazuwa-Peters

**Affiliations:** 1 Yale College, Yale University, New Haven, Connecticut; 2 REACH Equity Summer Undergraduate Research Program, Duke University School of Medicine, Durham, North Carolina; 3 Morehouse College, Atlanta, Georgia; 4 Department of Population Health Sciences, Duke University School of Medicine, Durham, North Carolina; 5 Division of Infectious Diseases, Department of Pediatrics, Duke University School of Medicine, Durham, North Carolina; 6 Duke Children’s Health and Discovery Initiative, Duke University School of Medicine, Durham, North Carolina; 7 Department of Radiation Oncology, Washington University School of Medicine in St. Louis, St Louis, Missouri; 8 Department of Head and Neck Surgery and Communication Sciences, Duke University School of Medicine, Durham, North Carolina; 9 Duke Cancer Institute, Duke University, Durham, North Carolina

## Abstract

**Importance:**

Depression and anxiety disproportionately impact cancer survivors. Sociodemographic factors frequently impact access to cancer care; however, it is unclear if these factors are associated with access to pharmacological care for mental and behavioral health conditions.

**Objective:**

To evaluate antidepressant and anxiolytic use among cancer survivors compared with the general US population and identify associations between sociodemographic factors and medication use.

**Design, Setting, and Participants:**

For this cross-sectional study, data from 2016 to 2018 from the National Health Interview Survey were analyzed in January 2024. The nationally representative, population-based sample consisted of respondents to the National Health Interview Survey aged 18 to 85 years without a history of cancer residing in the US.

**Main outcomes and measures:**

Eligible respondents completed the Adult Functioning and Disability supplements and had nonmissing outcomes. Self-reported antidepressant and anxiolytic use were the main outcomes, estimated through multivariable logistic regression analyses, adjusting for sociodemographic factors.

**Results:**

Among 53 117 respondents, 48 026 individuals (21 592 aged 40-64 years [41.8%]; 24 589 female [51.2%]; 8260 Hispanic [17.2%], 5859 non-Hispanic Black [12.2%], and 29 584 non-Hispanic White [61.6%]) reported no history of cancer and 5091 individuals (1624 aged 40-64 years [37.7%]; 2927 female [57.5%]; 321 Hispanic [6.3%], 361 non-Hispanic Black [7.1%], and 4159 non-Hispanic White [81.7%]) reported a history of cancer. After adjustment for sociodemographic variables, cancer survivors were more likely to report taking antidepressants (adjusted odds ratio [aOR], 1.32; 95% CI, 1.18-1.49) and anxiolytics (aOR, 1.38; 95% CI, 1.23-1.54) compared with the general population. Among cancer survivors, non-Hispanic Black individuals had lower odds of taking antidepressants (aOR, 0.60; 95% CI, 0.39-0.91) and anxiolytics (aOR, 0.63; 95% CI, 0.42-0.94) compared with non-Hispanic White individuals. Cancer survivors on Medicare (aOR,  2.20; 95% CI, 1.39-3.50) or Medicaid (aOR, 1.83; 95% CI, 1.18-2.84) were more likely to take anxiolytics compared with privately insured individuals. Cancer type was also associated with medication use; respondents with a history of brain cancer were more likely to take antidepressants (aOR, 5.59; 95% CI, 1.79-17.46), and those with a history of pancreatic cancer were more likely to take antidepressants (aOR, 5.30; 95% CI, 1.64-17.18) and anxiolytics (aOR, 6.74; 95% CI, 2.11-21.55) compared with those with a history of breast cancer.

**Conclusions and Relevance:**

In this study, cancer survivors were significantly more likely to take medications for depression and anxiety compared with noncancer survivors, underscoring the importance of mental health in cancer. However, our findings suggested disparities associated with this, with non-Hispanic Black patients exhibiting decreased use.

## Key Points

**Question:**

Are sociodemographic characteristics associated with antidepressant and anxiolytic use in cancer survivors?

**Findings:**

In this cross-sectional study using national data that included 5091 adult cancer survivors, non-Hispanic Black respondents had decreased odds of antidepressant use and anxiolytic use compared with non-Hispanic White cancer survivors, a significant difference. There were also significant differences by cancer type.

**Meaning:**

While this study found that cancer survivors were more likely to use medication for depression and anxiety, disparities by race and ethnicity still existed in the use of these medications.

## Teaser

This cross-sectional study investigates the use of antidepressant and anxiolytic medications among adult cancer survivors overall and by sociodemographic characteristics.

## Introduction

Cancer diagnoses are typically associated with increased risk of mental health concerns, including depression and anxiety.^1,2,3,4,5^ Individuals with a history of cancer also have significantly increased risk of death by suicide compared with those with no history of cancer, and this rate has been increasing since 1973.^1,6,7,8^ While treatment effectiveness, cure, physical recovery, and survival are typically the focus of most cancer treatments, mental health is increasingly being recognized as critical to long-term outcomes in cancer survivors.^9,10^ Across cancer types, patients with major depression have poorer survival and overall outcomes.^11,12,13,14,15,16^ With more than 18 million cancer survivors currently living in the US,^17^ evaluating and treating the mental health of patients with cancer during and after their treatment is critical.

Due to the nature of a cancer diagnosis, it is understandable that patients experience distress due to both the physical impact of the illness and the emotional burden it imposes. Depression and anxiety in cancer survivors may be a situational response to their diagnosis or a preexisting mental health condition. Some research suggests a bidirectional relationship, with depression, anxiety, or both potentially contributing to the development of cancer.^18,19,20^ Despite these complexities, medications remain an effective and safe method to manage anxiety and depression in the cancer survivor population.^21,22,23,24^

As of 2013, 16.8% of cancer survivors reported taking medicine for anxiety and 14.1% took medicine for depression, almost twice the rate in populations with no history of cancer.^25^ Pharmacological treatments and psychotherapy are available options to individuals experiencing depression and anxiety. Among available psychotherapies, group and individual cognitive behavioral therapy, as well as psychoeducation have been shown to improve depression and anxiety symptoms in the short term for patients with cancer.^26,27,28^ However, studies suggest that collaborative care combining pharmacotherapy and psychotherapy may be more beneficial for symptoms and quality of life than either approach alone among cancer survivors with depression.^29,30^ Despite availability of effective treatment combinations, there are significant inequities in access to mental health care in the US.^31^

Our study aimed to identify sociodemographic factors associated with pharmacological management of depression and anxiety among individuals with a history of cancer. We hypothesized that the prevalence of antidepressant and anxiolytic use in cancer survivors would differ across racial and ethnic groups when controlling for sociodemographic factors. To test our hypothesis, we examined associations between sociodemographic characteristics and medication use for depression or anxiety among cancer survivors.

## Methods

### Data Source

Analyses for this cross-sectional study were conducted in January 2024 using data from the National Health Interview Survey (NHIS) collected from 2016 to 2018. This cross-sectional survey collects self-reported data from US households annually.^32^ Due to survey restructuring in 2019 and lower response rates in the years after the COVID-19 pandemic, we chose to evaluate data from 2016 to 2018. Additionally, new supplemental questions are added each year, and these may or may not be kept the following year. Due to the change in study design, we chose to use a 3-year period for consistency and diversity of questions. Within each year, we combined NHIS Person and Adult files, as well as Adult Functioning and Disability (AFD) supplement files in 2016 and 2017, then further pooled combined data files together across years. Patient informed consent and further institutional review and approval were not required for this study because the NHIS dataset is publicly available and deidentified, making this study exempt from institutional review board approval. The results are reported following the Strengthening the Reporting of Observational Studies in Epidemiology (STROBE) reporting guideline reporting guideline for cross-sectional studies.

### Study Sample

The study sample consisted of adults between 18 and 85 years of age, residing in the United States. NHIS excludes individuals living in correctional facilities, long-term care institutions, or foreign countries or on military bases and anyone without a fixed household address. Eligible respondents for our study included 4 individuals who completed AFD supplements in 2016 and 2017 and all respondents in 2018 given that they all completed the AFD supplement in that year. Eligible respondents were required to have nonmissing outcomes, and respondents with nonmelanoma skin cancer were excluded.

### Outcomes

There were 2 primary outcomes. The first was antidepressant use, derived based on the survey question “Do you take medication for depression?” The second was anxiolytic use, derived based on the survey question “Do you take medication for anxiety?” Respondents could select 1 of 5 responses: “Yes,” “No,” “Refused,” “Not ascertained,” and “Don’t know.” A binary variable was created for each outcome, in which respondents who indicated *yes* to the question of interest were flagged as a 1 and all other respondents were flagged as a 0.

### Measures

#### Sociodemographic Characteristics

Sociodemographic characteristics analyzed were race and ethnicity, age, sex, marital status, income, education, insurance, geographic region of the US, year of survey, length of residence in the US, and English proficiency. Race and ethnicity were self-reported. Available race categories were American Indian or Alaska Native, Asian, Black, White, and mixed, other race, multiple races, and race groups not releasable. Ethnicity categories were Hispanic or non-Hispanic. Race categories are given for non-Hispanic respondents only. Due to sample size considerations, we combined all other racial and ethnic groups except Hispanic, non-Hispanic Black, and non-Hispanic White as other.

#### Cancer History

**Figure 1.**
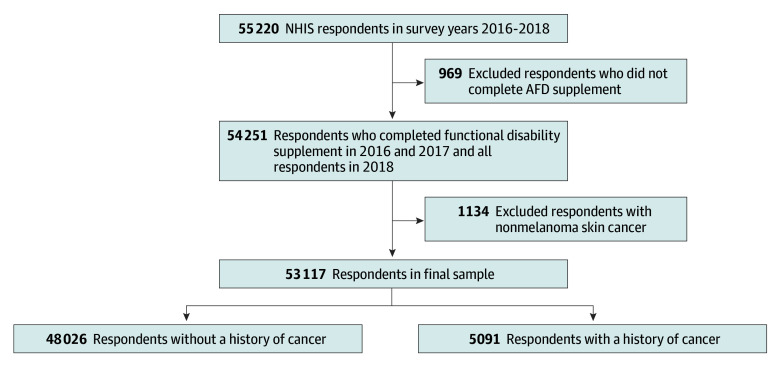
Study Flowchart The flowchart presents a breakdown on how final sample sizes were achieved for the full study cohort (all respondents who satisfied inclusion criteria) and subcohorts (by history of cancer). AFD indicates Adult Functioning and Disability; NHIS, National Health Interview Survey.

Cancer survivors included anyone who had been diagnosed with cancer at any point, including those undergoing cancer treatment when the survey was conducted. Individuals with a history of cancer were identified by a positive response to the question “Have you ever been told by a doctor or other health professional that you had cancer or a malignancy of any kind?” Response options for the cancer history variable were “Yes,” “No,” “Refused,” “Not ascertained,” and “Don’t know.” A binary variable was created for cancer history, with any respondent who indicated a yes flagged as a 1 and all others flagged as a 0. Among the cancer population, cancer type was grouped along the following categories: breast, brain, cervix, colorectal (colon or rectal), head and neck (larynx, mouth, or throat), hematologic (blood, leukemia, or lymphoma), lung, pancreas, prostate, multiple, and other or unknown. A study flowchart depicting the development of the cohort is shown in Figure 1.

### Statistical Analysis

Reports of antidepressant and anxiolytic use were analyzed in separate models. We first used univariate logistic regression analyses to assess for an association between cancer history and medication use for depression and for anxiety in the full study cohort. Next, we ran separate multivariable logistic regression models to examine the adjusted association between cancer history and each outcome, adjusting for age category, sex, marital status, race and ethnicity, income level, education, insurance status, geographical region, survey year, length of residence, and English language proficiency. Similarly, within the cancer history subcohort, we assessed associations between race and ethnicity and medication use for depression and for anxiety. We estimated adjusted associations using multivariable logistic regression models that included the same set of factors as for the full cohort, except that cancer type replaced cancer history. We report crude odds ratios (ORs) or adjusted ORs (aORs), with 95% CIs, as applicable. All analyses were adjusted for complex stratification and clustered sampling design of NHIS using sampling weights (AFD weight for 2016 and 2017 and sample adult weight for 2018) to obtain nationally representative estimates. Provided sampling weights were further adjusted by dividing by 3 given that 3 years of survey data were pooled together. Interactions between cancer history and sociodemographic variables were analyzed using type III analysis of variance (eTable 1 in Supplement 1), and significant interactions were stratified by subgroup (eTable 2 in Supplement 1). We estimated the proportion of respondents who selected a “Refused,” “Not ascertained,” or “Don’t know” in our study sample and conducted a sensitivity analyses that excluded these respondents (eTable 3 in Supplement 1). All *P* values are 2-sided, and we defined *P* < .05 as the threshold of statistical significance. Analyses were performed using R statistical software version 4.2.2 (R Project for Statistical Computing).^33^

## Results

**Figure 2.**
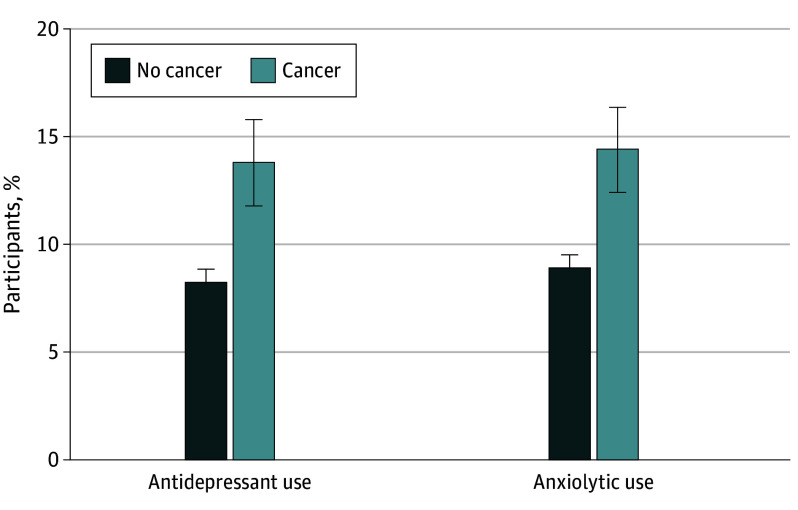
Use of Anxiolytic or Antidepressant Medications by Cancer Status Use is shown as weighted proportions and SEs (error bars) expressed as percentages. Data are from the National Health Interview Survey, 2016 to 2018.

A total of 53 117 respondents were included in the study sample, among whom 48 026 individuals (21 592 aged 40-64 years [41.8%]; 8260 Hispanic [17.2%], 5859 non-Hispanic Black [12.2%], and 29 584 non-Hispanic White [61.6%]; 24 589 female [51.2%]) reported no history of cancer and 5091 individuals (1624 aged 40-64 years [37.7%]; 321 Hispanic [6.3%], 361 non-Hispanic Black [7.1%], and 4159 non-Hispanic White [81.7%]; 2927 female [57.5%]) reported a history of cancer (Table 1). Respondents with a history of cancer reported taking medications for depression and anxiety at almost double the rate of respondents without a history of cancer; 748 respondents (13.8%) and 783 respondents (14.5%) with a history of cancer took medication for depression and anxiety, respectively, whereas 4551 respondents (8.3%) and 4838 respondents (8.9%) without a history of cancer took medication for depression and anxiety, respectively (Figure 2). In unadjusted models, respondents with a history of cancer had higher odds for antidepressant use (OR, 1.77; 95% CI, 1.60-1.97; *P* < .001) and anxiolytic use (OR, 1.72; 95% CI, 1.55-1.91; *P* < .001) compared with respondents without a history of cancer (eTable 4 in Supplement 1). Fully adjusted estimates showed similar patterns, albeit with ORs of reduced magnitude (Table 2); respondents with a cancer history had an aOR of 1.32 for antidepressant use (95% CI, 1.18-1.49; *P* < .001) and an aOR of 1.38 for anxiolytic use (95% CI, 1.23-1.54; *P* < .001).

**Table 1.** Baseline Characteristics

Characteristic	Respondents, weighted % (SE%) (N = 53 117)	
	No cancer (n = 48 026)	Cancer (n = 5091)
Age, y		
<40	41.6 (0.4)	6.4 (0.5)
40-64	41.8 (0.3)	37.7 (0.9)
65-74	10.3 (0.2)	28.5 (0.8)
75-84	4.5 (0.1)	19.8 (0.7)
≥85	1.8 (0.1)	7.6 (0.4)
Race and ethnicity		
Hispanic	17.2 (0.7)	6.3 (0.6)
Non-Hispanic Black	12.2 (0.4)	7.1 (0.6)
Non-Hispanic White	61.6 (0.8)	81.7 (0.9)
Other^a^	9.0 (0.4)	4.8 (0.5)
Sex		
Male	48.8 (0.3)	42.5 (0.9)
Female	51.2 (0.3)	57.5 (0.9)
Marital status		
Married	59.7 (0.4)	63 (0.9)
Not married	15.9 (0.2)	29.1 (0.8)
Never married	24.3 (0.3)	7.9 (0.5)
Unknown	0.2 (<0.1)	<0.1 (<0.1)
Length of residence, y		
US born	80.1 (0.6)	91 (0.6)
<10	3.8 (0.2)	0.4 (0.1)
≥10	15.0 (0.5)	8.0 (0.6)
Unknown	1.1 (0.1)	0.5 (0.1)
Geographic region		
South	36.2 (1.0)	34.8 (1.3)
Northeast	17.9 (0.7)	19.1 (1.1)
Midwest	21.9 (0.7)	24.2 (1.0)
West	24.0 (0.9)	22.0 (1.3)
Education		
≤High school	36.5 (0.5)	36.3 (0.9)
Some college or associate’s degree	30.5 (0.4)	30.7 (0.8)
≥College graduate	32.6 (0.5)	32.4 (1)
Unknown	0.4 (<0.1)	0.6 (0.2)
Insurance status		
Private	57.8 (0.4)	31.1 (0.9)
Uninsured	10.8 (0.3)	3 (0.3)
Medicare	18.5 (0.3)	58.8 (0.9)
Medicaid	9.7 (0.3)	5.3 (0.4)
Other	3.3 (0.1)	1.8 (0.3)
English proficiency		
Very good or good	94.2 (0.3)	97.7 (0.3)
Not good or none	5.8 (0.3)	2.3 (0.3)
Unknown	<0.1 (<0.1)	<0.1 (<0.1)
Year		
2016	32.6 (0.4)	33.2 (1)
2017	33 (0.4)	33.2 (1)
2018	34.4 (0.4)	33.6 (0.9)
Cancer type		
Breast	<0.1 (<0.1)	17.7 (0.7)
Brain	<0.1 (<0.1)	0.5 (0.1)
Cervix	<0.1 (<0.1)	6.1 (0.5)
Colorectal	<0.1 (<0.1)	6.1 (0.5)
Head and neck	<0.1 (<0.1)	1.5 (0.2)
Hematologic	<0.1 (<0.1)	5.2 (0.4)
Lung	<0.1 (<0.1)	2 (0.3)
Pancreas	<0.1 (<0.1)	0.4 (0.1)
Prostate	<0.1 (<0.1)	12.1 (0.6)
Nonmelanoma skin	<0.1 (<0.1)	<0.1 (<0.1)
Multiple	<0.1 (<0.1)	13.7 (0.6)
Other or unknown	<0.1 (<0.1)	34.7 (0.9)
None	100 (<0.1)	<0.1 (<0.1)

^a^ Other race was defined as American Indian or Alaska Native, Asian, or mixed, other race, multiple races, or race groups not releasable.

**Table 2.** Adjusted Association Between Cancer Status and Medication Use Among All Respondents (N = 53 117)

Characteristic	Medication for depression		Medication for anxiety	
	aOR (95% CI)	*P* value	aOR (95% CI)	*P* value
Cancer (reference group, no cancer)	1.32 (1.18-1.49)	<.001	1.38 (1.23-1.54)	<.001
Age (reference group, <40), y				
40-64	1.53 (1.37-1.71)	<.001	1.35 (1.21-1.51)	<.001
65-74	0.37 (0.30-0.45)	<.001	0.32 (0.26-0.39)	<.001
75-84	0.23 (0.18-0.30)	<.001	0.22 (0.17-0.28)	<.001
≥85	0.16 (0.12-0.22)	<.001	0.15 (0.11-0.21)	<.001
Female sex (reference group, male sex)	1.83 (1.68-2.00)	<.001	1.85 (1.7-2.01)	<.001
Marital status (reference group, married)				
Not married	1.53 (1.39-1.68)	<.001	1.35 (1.23-1.49)	<.001
Never married	1.24 (1.10-1.4)	<.001	1.17 (1.04-1.31)	.008
Unknown	0.36 (0.12-1.09)	.07	0.39 (0.15-1.02)	.05
Race and ethnicity (reference group, non-Hispanic White)				
Hispanic	0.59 (0.50-0.70)	<.001	0.60 (0.51-0.70)	<.001
Non-Hispanic Black	0.38 (0.33-0.44)	<.001	0.37 (0.32-0.44)	<.001
Other	0.50 (0.41-0.61)	<.001	0.61 (0.50-0.74)	<.001
Annual income (reference group, >75 000), $				
<45 000	1.56 (1.32-1.85)	<.001	1.43 (1.21-1.69)	<.001
45 000 to <75 000	1.58 (1.30-1.92)	<.001	1.58 (1.33-1.88)	<.001
Unknown	2.03 (1.72-2.39)	<.001	1.78 (1.51-2.10)	<.001
Education(reference group, ≤high school)				
Some college or associate’s degree	1.12 (1.02-1.22)	.02	1.16 (1.07-1.27)	.001
≥College	1.10 (0.99-1.22)	.07	1.05 (0.95-1.17)	.30
Unknown	0.52 (0.28-0.96)	.04	1.04 (0.56-1.93)	.99
Insurance (reference group, private)				
Uninsured	0.73 (0.60-0.90)	.003	0.65 (0.54-0.79)	<.001
Medicare	4.13 (3.51-4.85)	<.001	3.65 (3.08-4.34)	<.001
Medicaid	2.56 (2.23-2.94)	<.001	2.18 (1.91-2.49)	<.001
Other	1.77 (1.43-2.20)	<.001	1.54 (1.26-1.90)	<.001
Region (reference group, South)				
Northeast	0.87 (0.76-0.98)	.03	0.89 (0.78-1.00)	.06
Midwest	1.01 (0.91-1.13)	.83	0.99 (0.89-1.1)	.91
West	0.88 (0.77-1.01)	.07	0.81 (0.71-0.92)	.001
Year (reference group, 2016)				
2017	1.06 (0.95-1.18)	.28	1.12 (1.01-1.25)	.03
2018	1.18 (1.07-1.30)	.001	1.27 (1.16-1.4)	<.001
Length of residence (reference group, US born), y				
<10	0.31 (0.20-0.50)	<.001	0.27 (0.17-0.42)	<.001
≥10	0.61 (0.51-0.74)	<.001	0.58 (0.48-0.70)	<.001
Unknown	0.99 (0.63-1.55)	.97	0.92 (0.59-1.45)	.72
English proficiency (reference group, very good or good)				
Not good or none	0.98 (0.77-1.25)	.87	0.98 (0.75-1.29)	.91
Unknown	0.00 (0.00-0.00)	<.001	0.00 (0.00-0.00)	<.001

Abbreviation: aOR, adjusted odds ratio.

We next examined whether sociodemographic factors were associated with use of antidepressants or anxiolytics among individuals with a history of cancer (see eFigure and eTable 5 in Supplement 1 for baseline characteristics of individuals with a history of cancer by race and ethnicity). In fully adjusted multivariable models, non-Hispanic Black respondents were less likely to take antidepressants compared with non-Hispanic White respondents (aOR, 0.60; 95% CI, 0.39-0.91; *P* = .02) (Table 3). A similar pattern was observed for anxiolytic use, with decreased odds for non-Hispanic Black respondents to use anxiolytics compared with non-Hispanic White respondents (aOR, 0.63; 95% CI, 0.42-0.94; *P* = .03) (Table 3).

**Table 3.** Adjusted Associations Between Study Covariates and Medication Use Among Respondents With History of Cancer (n = 5091)

Characteristic	Medication for depression		Medication for anxiety	
	aOR (95% CI)	*P* value	aOR (95% CI)	*P* value
Age (reference group, <40) y				
40-64	1.17 (0.75-1.82)	.48	0.78 (0.50-1.20)	.25
65-74	0.26 (0.15-0.45)	<.001	0.23 (0.13-0.41)	<.001
75-84	0.17 (0.09-0.31)	<.001	0.15 (0.08-0.28)	<.001
≥85	0.08 (0.04-0.16)	<.001	0.08 (0.04-0.15)	<.001
Female sex (reference group, male sex)	1.70 (1.28-2.25)	<.001	1.57 (1.19-2.08)	.002
Marital status (reference group, married)				
Not married	1.45 (1.15-1.82)	.002	1.41 (1.12-1.76)	.003
Never married	1.49 (1.02-2.19)	.04	1.22 (0.84-1.78)	.299
Unknown	0.00 (0.00-0.00)	<.001	0.00 (0.00-0.00)	<.001
Race and ethnicity (reference group, non-Hispanic White)				
Hispanic	0.92 (0.54-1.57)	.76	1.06 (0.63-1.78)	.84
Non-Hispanic Black	0.6 (0.39-0.91)	.02	0.63 (0.42-0.94)	.03
Other	0.72 (0.4-1.3)	.28	0.94 (0.54-1.62)	.83
Annual income (reference group, >75 000), $				
<45 000	1.11 (0.60-2.06)	.74	1.20 (0.68-2.12)	.53
45 000 to <75 000	1.31 (0.66-2.60)	.45	1.09 (0.57-2.06)	.80
Unknown	1.71 (0.93-3.13)	.08	2.08 (1.21-3.60)	.009
Education (reference group, ≤high school)				
Some college or associate’s degree	0.86 (0.67-1.09)	.22	0.94 (0.75-1.19)	.62
≥College	0.90 (0.70-1.16)	.41	0.84 (0.64-1.10)	.20
Unknown	1.35 (0.34-5.33)	.67	1.81 (0.42-7.74)	.42
Insurance (reference group, private)				
Uninsured	1.04 (0.52-2.09)	.91	0.68 (0.38-1.22)	.20
Medicare	4.36 (2.88-6.62)	<.001	2.20 (1.39-3.50)	.001
Medicaid	2.64 (1.67-4.18)	<.001	1.83 (1.18-2.84)	.007
Other	1.67 (0.78-3.60)	.19	1.37 (0.68-2.77)	.38
Region (reference group, South)				
Northeast	1.19 (0.89-1.59)	.25	0.92 (0.69-1.23)	.58
Midwest	1.06 (0.81-1.39)	.66	0.97 (0.76-1.26)	.84
West	0.93 (0.69-1.26)	.66	0.69 (0.51-0.94)	.02
Year (reference group, 2016)				
2017	1.07 (0.80-1.42)	.65	1.20 (0.91-1.57)	.20
2018	1.23 (0.95-1.60)	.116	1.33 (1.03-1.71)	.03
Length of residence (reference group, US born), y				
<10	0.00 (0.00-0.00)	<.001	0.37 (0.04-3.12)	.36
≥10	0.87 (0.51-1.48)	.61	0.76 (0.45-1.28)	.30
Unknown	0.17 (0.02-1.50)	.11	0.00 (0.00-0.00)	<.001
English proficiency (reference group, very good or good)				
Not good or none	0.72 (0.30-1.72)	.46	0.45 (0.18-1.12)	.09
Cancer type (reference group, breast)				
Brain	5.59 (1.79-17.46)	.003	2.38 (0.54-10.50)	.25
Cervix	1.48 (0.96-2.28)	.07	1.47 (0.93-2.31)	.10
Colorectal	1.39 (0.77-2.49)	.27	1.43 (0.84-2.44)	.19
Head and neck	1.25 (0.48-3.27)	.65	1.56 (0.64-3.80)	.33
Hematologic	1.10 (0.59-2.05)	.77	1.67 (0.99-2.82)	.06
Lung	2.08 (0.94-4.58)	.07	1.69 (0.78-3.66)	.19
Pancreas	5.30 (1.64-17.18)	.006	6.74 (2.11-21.55)	.001
Prostate	1.43 (0.88-2.32)	.15	1.25 (0.76-2.06)	.39
Multiple	1.05 (0.73-1.52)	.80	1.25 (0.87-1.79)	.22
Other or unknown	1.20 (0.88-1.64)	.26	1.15 (0.84-1.57)	.39

Abbreviation: aOR, adjusted odds ratio.

We additionally observed differences in the likelihood of medication use by insurance status and marital status (Table 3). There were higher odds for antidepressant use among respondents with Medicare (aOR, 4.36; 95% CI, 2.88-6.62; *P* < .001) and Medicaid (aOR, 2.64; 95% CI, 1.67-4.18; *P* < .001) compared with privately insured respondents. Similarly, respondents covered by Medicare (aOR, 2.20; 95% CI, 1.39-3.50; *P* = .001) and Medicaid (aOR, 1.83; 95% CI, 1.18-2.84; *P* = .007) were more likely to take anxiolytics compared with privately insured respondents.

We additionally observed that cancer survivors who were not married were more likely to report taking antidepressants (aOR, 1.45; 95% CI, 1.15-1.82; *P* = .002) and anxiolytics (aOR, 1.41; 95% CI, 1.13-1.17; *P* = .003) compared with married cancer survivors. Respondents who were never married were more likely to report taking antidepressants (aOR, 1.49; 95% CI, 1.02-2.19; *P* = .04) compared with those who were married.

Cancer types were differentially associated with use of antidepressants and anxiolytics (Table 3). There was an increase in the odds of taking antidepressants among respondents with a history of brain cancer (aOR, 5.59; 95% CI, 1.79-17.46; *P* = .003) and pancreatic cancer (aOR, 5.30; 95% CI, 1.64-17.18; *P* = .006) compared with respondents with a history of breast cancer. Similarly, respondents with a history of pancreatic cancer had increased odds of anxiolytic use compared with respondents with a history of breast cancer (aOR, 6.74; 95% CI, 2.11-21.55; *P* = .001).

## Discussion

The objective of this cross-sectional study was to investigate associations between social determinants of health and use of depression and anxiety medication among cancer survivors using a nationally representative dataset. We hypothesized that antidepressant and anxiolytic use among cancer survivors would be significantly different based on sociodemographic factors. Our hypothesis was supported by results showing that Black cancer survivors were significantly less likely to use antidepressants and anxiolytics compared with White cancer survivors. We also noted differences based on health insurance and other factors. By highlighting sociodemographic characteristics associated with differential use of medications for depression and anxiety among cancer survivors, this study adds to the literature on cancer survivorship and quality of care.

Our findings were consistent with those of previous studies indicating higher use rates of medications for depression and anxiety among cancer survivors compared with the general population.^24,25,34^ While depression and anxiety are prevalent in the general population, our study underscores the association of a cancer diagnosis with the mental health of patients with cancer throughout the treatment process and beyond. A cancer diagnosis has short- and long-term psychological impacts on cancer survivors across the cancer continuum, as well as on their families.^35,36,37,38^ Therefore, treatment for mental health must be a priority during and after cancer care.^1,39,40^

We found that non-Hispanic Black cancer survivors reported using antidepressants and anxiolytics at significantly lower rates than non-Hispanic White cancer survivors. This difference in mental health treatment rate by race and ethnicity is consistent with treatment disparities between non-Hispanic Black and non-Hispanic White individuals in the general US population, and although it was considerably smaller than disparities seen in the combined cohort data, the difference remained significant.^41^ A myriad of factors, such as stigma surrounding treatment, lack of trust in health care, clinician bias, and differential access to care, may contribute to this disparity among cancer survivors.^42,43,44^ Prior studies suggest that cultural beliefs and stigma toward mental health may play a role because Black individuals report higher rates of judgment directed toward individuals with mental health issues within their communities than White individuals.^45,46^ Additionally, non-Hispanic Black patients are less likely to have a racially concordant clinician,^47^ and racial discordance is associated with lower reported levels of trust.^48^ Black cancer survivors and other patients who are members of racial and ethnic minority groups are also less likely to have access to mental health care, including care for depression and anxiety.^49,50^ More research is needed to determine the direct impact of these factors on clinical outcomes, specifically on antidepressant and anxiolytic usage.

The role of social support in the mental health care of cancer survivors was illustrated in our finding of differential use of antidepressants and anxiolytics by marital status, with unmarried individuals significantly more likely to use antidepressants and anxiolytics. Marital status is a known proxy for social support and has been shown to have significant implications in cancer outcomes.^51,52,53^ While consistent with depression trends within the general population,^54^ these results support evidence that social support systems, such as the presence of a spouse, are associated with improved care and outcomes of cancer survivors.^52,55^ Several prior studies^56,57,58^ have demonstrated that marital status and social support systems are associated with improved survival and quality of life during and after treatment. It is important that oncology care pay needed attention to the mental health needs of cancer survivors who lack adequate social support.

Health insurance plays a critical role in access to cancer care and mental and behavioral health care. We found that patients with cancer on Medicare and Medicaid reported using antidepressants and anxiolytics at significantly higher rates than privately insured individuals. The increasing psychiatrist shortage and shift to providing medication management rather than psychotherapy may contribute to these findings.^59^ Compared with physicians from other specialties, psychiatrists are significantly less likely to accept Medicare and Medicaid.^59^ Furthermore, the main health care clinician after treatment for most cancer survivors is their primary care physician.^60^ Most primary care physicians report directly managing treatment for depression,^61^ and a higher number of primary care visits was associated with increased likelihood of psychotherapy use.^62^ Additionally, Tabriz et al^63^ found that more than half of visits to the emergency department made by patients with cancer were among Medicare enrollees.

### Public Health Implications

Social determinants of health play a role in health care access and quality in many areas, including the increasing mental health crisis within the general US population.^41^ While depression and anxiety are nationwide problems, they are especially prevalent among the cancer population. Due to the large impact that depression and anxiety have on survival of patients with cancer during treatment and beyond,^13^ mental health care for depression and anxiety should be a priority throughout cancer treatment. Disparities highlighted in this study provide insight into groups of cancer survivors that may be less likely to use pharmacological treatment for depression and anxiety and raise the question of whether the disparities arise from personal choice or a lack of opportunity.

In 2015, the American College of Surgeons mandated a cancer distress screening for Commission on Cancer accreditation.^64^ Based on the 2020 Standards and Resources, patients with cancer must be screened for distress at least once during their first course of treatment.^65^ While this is an important step forward, current regulations do not mandate screening for cancer survivors admitted for non–cancer-related issues, and the implementation of screenings in the clinic still would not account for cancer survivors who do not visit the clinic regularly.^66^ Standardizing and increasing screening frequency may be beneficial for all patients, especially cancer survivors. Continued integration of mental health specialists into cancer centers and better awareness and recognition of the impact of mental health on cancer outcomes could solidify mental health treatment as an essential part of cancer care and help eliminate observed stigma and disparities.^67,68^

### Strengths and Limitations

Our study has several strengths and limitations. Although we had a relatively large sample size, a limitation of our study was the limited diversity of our sample. The non-Hispanic White group made up 81.7% of the cancer cohort, with more than 4000 patients, whereas Hispanic and non-Hispanic Black groups combined consisted of fewer than 700 patients. This could explain the large margin of error for the non-Hispanic Black and Hispanic populations compared with the non-Hispanic White population.

Although we evaluated by cancer type, we did not distinguish by cancer stage, time after diagnosis, or cancer severity, which are factors that may contribute to depression and anxiety.^68,69^ Respondents with a history of cancer could be at any stage of treatment given that cancer survivors encompass a broad range of individuals, including those who were recently diagnosed, are currently undergoing treatment, and are in remission.^70^

Due to the nature of the survey questions, we did not have information about potential factors contributing to disparities present in our results. For example, we did not know if patients were referred to mental health services and could not specifically identify individuals who were prescribed antidepressants or anxiolytics but did not fill their prescriptions.

Our study was limited to examining the use of pharmacological treatment. Although nonpharmacological treatment options, such as psychological, educational, and exercise interventions, can improve depression and anxiety symptoms in patients with cancer,^71^ the analysis of these treatment options was beyond the scope of our study. Given the variables available in the dataset, we could not estimate how many patients were enrolled in psychotherapy for their depression or anxiety as opposed to or in addition to pharmacotherapy. A study looking at the general population^41^ found that Black patients treated for mental health (depression, anxiety, and other mood disorders) were more likely to receive psychotherapy but less likely to receive medications than White patients. More research is needed to understand if these differences in kinds of treatment are also present in the cancer survivor population. Evidence suggests that pharmacological treatment in combination with psychological interventions and active collaboration with clinicians can be the most effective way to treat depression and anxiety.^29,30^ Therefore, even if survivors were participating only in talk therapy, if they were at low odds for also using pharmacological treatment, their quality of care may be lower than if this disparity was not present.

Despite these limitations, this is among the largest nationally representative studies to date to focus on the association of social determinants of health with antidepressant and anxiolytic use among cancer survivors. Additionally, our study’s inclusion of a wide variety of sociodemographic variables is beneficial from an investigative standpoint. These variables provided parameters to adjust for, which may have mitigated their influence on each other, leading to a truer representation of each sociodemographic variable’s impact.

## Conclusions

This cross-sectional study of anxiolytic and antidepressant use among cancer survivors found that Black cancer survivors were less likely to use medications for depression and anxiety compared with White survivors after controlling for other sociodemographic characteristics. Given the potential impact that depression and anxiety can have on treatment outcomes, it is important to ensure equitable access to mental health for all cancer survivors independent of sociodemographic differences.

## Notes

Supplement 1. **eFigure.** Medication frequency by race and ethnicity for respondents with a history of cancer (unadjusted) **eTable 1.** Test of interactions between cancer history and sociodemographic variables using type III ANOVA test **eTable 2.** Stratified estimates of the association between cancer history and each outcome for each race and ethnicity subgroup separately **eTable 3.** Sensitivity analyses only including participants who responded to the outcome questions with yes or no (N = 52 128) **eTable 4.** Crude estimates of association between antidepressant and anxiolytic use and cancer status **eTable 5.** Baseline characteristics of respondents with a history of cancer by race and ethnicity

Supplement 2.
Data Sharing Statement
