# Supplementary material for: Differential Use of Depression and Anxiety Medications in Adults With a History of Cancer
Source: JAMA Netw Open. 2025 Aug 19;8(8):e2527585. doi: 10.1001/jamanetworkopen.2025.27585 (PMC12365704; doi:10.1001/jamanetworkopen.2025.27585)
Supplement: Supplement 1. — eFigure. Medication frequency by race and ethnicity for respondents with a history of cancer (unadjusted) eTable 1. Test of interactions between cancer history and sociodemographic variables using type III ANOVA test eTable 2. Stratified estimates of the association between cancer history and each outcome for each race and ethnicity subgroup separately eTable 3. Sensitivity analyses only including participants who responded to the outcome questions with yes or no (N = 52 128) eTable 4. Crude estimates of association between antidepressant and anxiolytic use and cancer status eTable 5. Baseline characteristics of respondents with a history of cancer by race and ethnicity [file jamanetwopen-e2527585-s001.pdf]

Supplemental Online Content

Miro-Rivera D, Norris RA, Osazuwa-Peters OL, Hurst JH, Barnes JM, Osazuwa-Peters N. Differential use of depression and anxiety medications in adults with a history of cancer. *JAMA Netw Open*. 2025;8(8):e2527585.
doi:10.1001/jamanetworkopen.2025.27585

eFigure. Medication frequency by race and ethnicity for respondents with a history of cancer (unadjusted)

eTable 1. Test of interactions between cancer history and sociodemographic variables using type III ANOVA test

eTable 2. Stratified estimates of the association between cancer history and each outcome for each race and ethnicity subgroup separately

eTable 3. Sensitivity analyses only including participants who responded to the outcome questions with yes or no (N = 52 128)

eTable 4. Crude estimates of association between antidepressant and anxiolytic use and cancer status

eTable 5. Baseline characteristics of respondents with a history of cancer by race and ethnicity

This supplemental material has been provided by the authors to give readers additional information about their work.

© 2025 Miro-Rivera D et al. *JAMA Network Open*.

eFigure. Medication frequency by race for respondents with a history of cancer (unadjusted); shown as weighted proportions and standard errors (error bars) expressed as percentages. National Health Interview Survey 2016 to 2018.

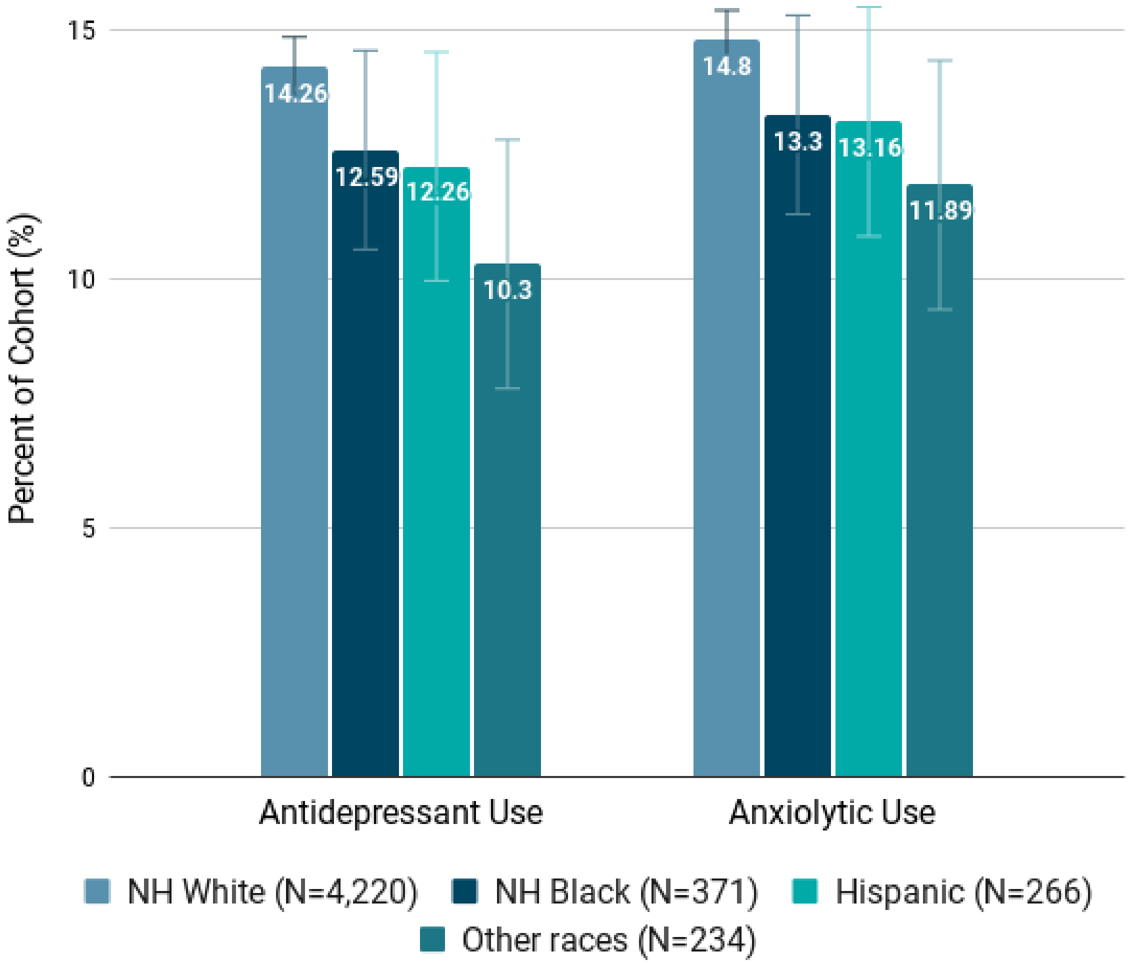

© 2025 Miro-Rivera D et al. *JAMA Network Open*.

eTable 1. Test of interactions between cancer history and sociodemographic variables using Type III ANOVA test.

Variable interacting with Cancer History	Outcome	Chisq	Df	P-value
Race and ethnicity	**Anxiety**	**14.89**	**3**	**1.9e-03**
Income	Anxiety	3.16	3	0.37
Race and ethnicity	**Depression**	**11.81**	**3**	**8.1e-03**
Income	Depression	1.25	3	0.74
**Note**: Significant P values at *P* < 0.05 are highlighted in bold				

eTable 2. Stratified estimates of the association between cancer history and each outcome for each race and ethnicity subgroup separately.

	Antidepressant Use		Anxiolytic Use	
**Race and ethnicity**	**aOR (95% CI)**	***P***	**aOR (95% CI)**	***P***
NH Black	**1.67 (1.1,2.53)**	**0.016**	**2.04 (1.34,3.1)**	**9e-04**
NH White	**1.3 (1.15,1.47)**	**5e-05**	**1.34 (1.18,1.51)**	**3e-06**
Hispanic	1.45 (0.88,2.4)	0.148	**1.71 (1.05,2.77)**	**0.031**
Others	2.17 (1.14,4.13)	0.018	1.74 (0.94,3.24)	0.08
**Note**: Significant P values at *P* < 0.05 are highlighted in bold				

eTable 3. Sensitivity analyses only including participants who responded to the outcome questions with yes or no (N=52,128)

	Medications for Depression		Medications for Anxiety	
**Variable**	**aOR (95% CI)**	***P***	**aOR (95% CI)**	***P***
Cancer status: Cancer vs No cancer	1.33 (1.19,1.49)	1.0e-06	1.38 (1.24,1.55)	1.6e-08
Age category: vs < 40 years				
40-64 years	1.52 (1.36,1.7)	2.9e-13	1.35 (1.21,1.51)	7.2e-08
65-74 years	0.36 (0.3,0.44)	1.5e-21	0.32 (0.26,0.39)	1.3e-26
75-84 years	0.23 (0.18,0.29)	6.6e-30	0.22 (0.17,0.28)	4.5e-31
85+ years	0.16 (0.12,0.22)	3.2e-28	0.15 (0.11,0.21)	1.6e-30
Sex: Female vs. Male	1.84 (1.69,2.01)	5.8e-38	1.86 (1.71,2.02)	1.2e-40
Marital status: vs Married				
Not married	1.54 (1.4,1.7)	2.4e-18	1.37 (1.24,1.5)	2.9e-10
Never married	1.25 (1.11,1.4)	3.2e-04	1.17 (1.04,1.32)	7.7e-03
Unknown	0.38 (0.12,1.14)	0.083	0.41 (0.16,1.06)	0.067
Race and ethnicity: vs NH White				
NH Black	0.38 (0.32,0.44)	8.2e-33	0.37 (0.32,0.44)	1.3e-31
Hispanic	0.58 (0.49,0.69)	1.5e-09	0.59 (0.5,0.69)	2.3e-10
Others	0.5 (0.41,0.61)	4.4e-11	0.61 (0.5,0.74)	9.5e-07
Income: vs >$75K				
Income: <$45K	1.56 (1.32,1.85)	3.2e-07	1.43 (1.21,1.69)	3.1e-05

© 2025 Miro-Rivera D et al. *JAMA Network Open*.

$45K-<$75K	1.58 (1.3,1.92)	4.0e-06	1.58 (1.33,1.88)	2.4e-07
Unknown	2.06 (1.75,2.43)	6.3e-17	1.81 (1.53,2.13)	5.6e-12
Education: vs High school or less				
Some college/associate degree	1.11 (1.01,1.21)	0.023	1.16 (1.06,1.27)	9.1e-04
College or higher	1.1 (0.99,1.22)	0.073	1.05 (0.95,1.16)	0.319
Unknown	0.49 (0.26,0.93)	0.03	1.04 (0.55,1.99)	0.9
Insurance: vs Private				
Uninsured	0.73 (0.6,0.9)	2.5e-03	0.65 (0.54,0.79)	1.1e-05
Medicare	4.15 (3.53,4.88)	4.2e-54	3.66 (3.08,4.35)	4.3e-42
Medicaid	2.56 (2.22,2.95)	9.9e-35	2.19 (1.92,2.5)	4.6e-28
Others	1.78 (1.43,2.21)	2.7e-07	1.54 (1.25,1.9)	4.9e-05
Region: vs South				
Northeast	0.87 (0.77,0.99)	0.031	0.89 (0.78,1.01)	0.073
Midwest	1.01 (0.9,1.13)	0.869	1 (0.9,1.11)	0.936
West	0.89 (0.78,1.01)	0.071	0.81 (0.72,0.92)	9.6e-04
Year: vs 2016				
2017	1.06 (0.96,1.18)	0.254	1.13 (1.01,1.25)	0.025
2018	1.22 (1.1,1.34)	8.1e-05	1.31 (1.19,1.44)	2.1e-08
Length of residence: vs US Born				
< 10 yrs	0.31 (0.2,0.5)	9.2e-07	0.27 (0.17,0.42)	2.1e-08
10 yrs or more	0.61 (0.51,0.75)	1.2e-06	0.59 (0.49,0.71)	2.4e-08
Unknown	1.02 (0.65,1.61)	0.922	0.96 (0.61,1.51)	0.852
English proficiency: vs Very good/good				
Not good/none	0.99 (0.78,1.26)	0.934	0.99 (0.75,1.29)	0.915
Unknown	0 (0,0)	1.5e-24	0 (0,0)	4.5e-26

Note: compare these results to Table 2 in the main text. There is no major difference when individuals who did not respond with a yes or no to the outcome questions were excluded.
Only about 3.5% of the original sample did not responded with 7 (refused), 8 (not ascertained), or 9 (don't know) to outcome questions; for depression medications there were 1,939 respondents, while for anxiety medications there were 1,903 who did not indicate a yes or no to outcome questions. Individuals that fell into this group for either anxiety or depression medications were excluded in this sensitivity analyses, resulting in a sample size of 52,128, compared to the original study sample of 53,117.

eTable 4. Crude estimates of association between antidepressant and anxiolytic use and cancer status.

Antidepressant Use		Anxiolytic Use	
**OR (95% CI)**	***P*-value**	**OR (95% CI)**	***P*-value**
1.77 (1.6-1.97)	<0.001	1.72 (1.55-1.91)	<0.001

© 2025 Miro-Rivera D et al. *JAMA Network Open*.

eTable 5. Baseline characteristics of respondents with a history of cancer by race and ethnicity, National Health Interview Survey 2016 to 2018; weighted proportions and standard error in parentheses expressed as percentages.

Characteristic	NH White (N=4,220)	NH Black (N=371)	Hispanic (N=266)	Other races (N=234)
Age				
<40 years	5.9 (0.5)	8 (2)	13.1 (2.7)	3.9 (1.4)
40-64 years	37.2 (1)	35.3 (3.2)	42.8 (3.3)	42.6 (4.2)
65-74 years	28.2 (0.9)	36.4 (3.5)	19.6 (2.9)	32.1 (3.8)
75-84 years	20.6 (0.8)	16.8 (2.4)	15.4 (2.4)	16.3 (2.8)
85+ years	8 (0.5)	3.6 (0.9)	9.1 (2.3)	5 (1.4)
Sex				
Male	42.3 (1)	45.9 (3.6)	42.5 (3.6)	40.4 (3.8)
Female	57.7 (1)	54.1 (3.6)	57.5 (3.6)	59.6 (3.8)
Marital status				
Married	65.2 (0.9)	42.5 (3.6)	53.6 (3.5)	68.9 (4.1)
Not married	27.6 (0.9)	39.7 (3.3)	38.2 (3.5)	25.9 (3.6)
Never married	7.2 (0.5)	17.7 (2.6)	8.2 (1.6)	5.2 (1.4)
Length of residence				
US born	96.6 (0.4)	90.7 (2.2)	44.1 (4)	58.7 (4.7)
< 10 years	0.1 (0)	0 (0)	2.1 (1.1)	4.5 (2.3)
10 years or more	3.2 (0.4)	9.1 (2.2)	47.8 (4.2)	36.1 (4.3)
Unknown	0.1 (0.1)	0.2 (0.2)	6.1 (1.5)	0.7 (0.5)
Geographical region				
South	32.8 (1.3)	60.4 (3.9)	38.7 (4.8)	24.4 (4)
Northeast	20.4 (1.2)	14.5 (2.4)	12.8 (3.1)	10.8 (2.6)
Midwest	26.5 (1.2)	17.6 (3)	8.2 (2.7)	15.5 (3.2)
West	20.3 (1.3)	7.4 (1.8)	40.3 (4.9)	49.4 (5.3)
Education				
High school or less	34.8 (1)	44.5 (3.6)	48.7 (3.7)	33.6 (4.1)
Some college/Associate degree	30.8 (0.9)	33.6 (3.2)	27.6 (3.2)	28.4 (3.4)
College graduate or higher	33.9 (1.1)	20.9 (2.9)	23.4 (3.3)	36.5 (4.4)
Unknown	0.5 (0.2)	1 (0.5)	0.2 (0.2)	1.5 (1.5)
Insurance status				
Private	32 (1)	20.5 (3.4)	30.6 (3.4)	32.8 (4.2)
Uninsured	2.5 (0.3)	3.2 (1.2)	8.2 (2.4)	5.2 (1.8)
Medicare	59.8 (1)	62.1 (3.5)	46.9 (3.7)	52 (4.4)
Medicaid	4.1 (0.4)	11.8 (2.4)	10.9 (2.5)	8.7 (2.5)
Others	1.6 (0.3)	2.3 (0.9)	3.4 (1.4)	1.3 (0.7)
English proficiency				
Very good/good	99.7 (0.1)	99.5 (0.3)	76.3 (3.8)	90.2 (2.8)
Not good/none	0.3 (0.1)	0.5 (0.3)	23.7 (3.8)	9.8 (2.8)
Year				
2016	33.3 (1.1)	31.2 (3.4)	34.1 (3.8)	32.5 (3.8)
2017	33.4 (1.1)	31.8 (3.8)	33.5 (3.6)	31 (4.5)
2018	33.3 (0.9)	36.9 (3.3)	32.4 (3)	36.5 (4.1)
Cancer type				
Breast	16.9 (0.7)	23.7 (2.9)	19.6 (2.9)	20.1 (3)
Brain	0.5 (0.2)	0.6 (0.6)	0.1 (0.1)	1 (0.9)
Cervix	6.1 (0.5)	6.3 (1.6)	5.2 (1.3)	7.1 (2.3)
Colorectal	5.9 (0.5)	5.5 (1.4)	7.1 (1.9)	9 (2.9)
Head and Neck	1.4 (0.2)	0.6 (0.4)	4.8 (1.6)	0.4 (0.3)
Hematologic	5.2 (0.5)	6.2 (1.6)	4.9 (1.4)	4.7 (1.8)

© 2025 Miro-Rivera D et al. *JAMA Network Open*.

Lung	1.8 (0.3)	3.4 (1.2)	2.2 (1.1)	3.8 (1.4)
Pancreas	0.4 (0.1)	0.4 (0.3)	0.5 (0.5)	0.3 (0.2)
Prostate	10.9 (0.6)	23.2 (2.8)	12.8 (2.6)	14.5 (3.1)
Non-melanoma skin	0 (0)	0 (0)	0 (0)	0 (0)
Multiple	14.5 (0.7)	9.9 (1.8)	13.3 (2.7)	4.9 (1.5)
Other/Unknown	36.5 (1)	20 (2.7)	29.5 (3.3)	34.2 (3.7)

© 2025 Miro-Rivera D et al. *JAMA Network Open*.
