# Supplementary material for: Differential Use of Depression and Anxiety Medications in Adults With a History of Cancer
Source: JAMA Netw Open. 2025 Aug 19;8(8):e2527585. doi: 10.1001/jamanetworkopen.2025.27585 (PMC12365704; doi:10.1001/jamanetworkopen.2025.27585)
Supplement: Supplement 2. — Data Sharing Statement [file jamanetwopen-e2527585-s002.pdf]

Data Sharing Statement

Miro-Rivera. Differential Use of Depression and Anxiety Medications in Adults With a History of Cancer. *JAMA Netw Open*. Published online August 19, 2025. doi:10.1001/jamanetworkopen.2025.27585

Data
Data available: No

Additional Information
Explanation for why data not available: The data from the National Health Interview Survey is publicly available.
